# Interpersonal problems and recognition of facial emotions in healthy individuals

**DOI:** 10.3389/fpsyt.2023.1139051

**Published:** 2023-04-17

**Authors:** Thomas Suslow, Alexander Lemster, Katja Koelkebeck, Anette Kersting

**Affiliations:** ^1^Department of Psychosomatic Medicine and Psychotherapy, University of Leipzig Medical Center, Leipzig, Germany; ^2^Department of Psychiatry and Psychotherapy, LVR-Hospital Essen, Institute and Hospital of the University of Duisburg-Essen, Essen, Germany

**Keywords:** interpersonal problems, agency, communion, emotion recognition, facial expression, anger, disgust

## Abstract

**Background:**

Recognition of emotions in faces is important for successful social interaction. Results from previous research based on clinical samples suggest that difficulties in identifying threat-related or negative emotions can go along with interpersonal problems. The present study examined whether associations between interpersonal difficulties and emotion decoding ability can be found in healthy individuals. Our analysis was focused on two main dimensions of interpersonal problems: agency (social dominance) and communion (social closeness).

**Materials and methods:**

We constructed an emotion recognition task with facial expressions depicting six basic emotions (happiness, surprise, anger, disgust, sadness, and fear) in frontal and profile view, which was administered to 190 healthy adults (95 women) with a mean age of 23.9 years (*SD* = 3.8) along with the Inventory of Interpersonal Problems, measures of negative affect and verbal intelligence. The majority of participants were university students (80%). Emotion recognition accuracy was assessed using unbiased hit rates.

**Results:**

Negative correlations were observed between interpersonal agency and recognition of facial anger and disgust that were independent of participants’ gender and negative affect. Interpersonal communion was not related to recognition of facial emotions.

**Discussion:**

Poor identification of other people’s facial signals of anger and disgust might be a factor contributing to interpersonal problems with social dominance and intrusiveness. Anger expressions signal goal obstruction and proneness to engage in conflict whereas facial disgust indicates a request to increase social distance. The interpersonal problem dimension of communion appears not to be linked to the ability to recognize emotions from facial expressions.

## Introduction

Interpersonal problems are recurrent difficulties that individuals have in relating to others (1). They are key constructs for understanding and characterizing psychopathology that cuts across diagnostic categories. Interpersonal relationship dysfunctions are encountered in a wide range of mental [e.g., social phobia, depressive, autism spectrum, and addictive disorders (2)] and personality disorders [e.g., borderline, paranoid, and avoidant personality disorder (3)]. Efforts to conceptualize interpersonal problems in the clinical context have relied primarily on the interpersonal circumplex, which is a model for assessing, and integrating interpersonal behaviors, traits, and motives (4). The interpersonal circumplex is defined by two orthogonal axes or dimensions, i.e., agency and communion (5). Agency refers to an individual’s control of others and includes traits such as dominance, assertiveness, and independence. It appears relevant to negotiating social hierarchies. Communion relates to involvement with others and includes traits such as caring, friendliness, and cooperation. Communion seems relevant to negotiating social distance. An important factor that could contribute to difficulties in interpersonal behavior and communication is an impaired recognition of emotions from facial expressions of other people (6). In mental disorders characterized by interpersonal problems such as major depression, autism spectrum disorder, or alcohol-related disorders substantial impairments in recognizing basic facial emotions have been documented [see for meta-analyses (7–9)].

Expression and recognition of emotions in the face are essential for social communication (10). Facial expressions of basic emotions such as happiness, surprise, fear, anger, sadness, and disgust are interpreted in similar ways across different cultures (11). The ability to accurately interpret others’ facial emotions is crucial in subsequently deciding on appropriate courses of action (12). Facial expressions do not only inform about the expresser’s emotional state (13), but they also allude to his or her appraisals, behavioral intentions, and action requests (14). Facial happiness, for example, can signal an invitation for approach and social interaction (15). Facial anger may indicate that a person experiences an obstruction of his/her goals and is convinced to have the capacity to cope with the problem and to assert his/her interests (16). The facial expressions of disgust can signal disapproval of others’ behaviors in social situations, and interpersonal rejection and can be perceived as socially threatening (17). Facial fear can be an indicator of danger or threat in the environment and the expresser’s weakness and loss of control (18).

Early research in preschool and elementary school children has revealed a link between the ability to correctly decode facial emotions and higher social skills (19) and increased popularity among classmates (20). In a study with young healthy adults (21), difficulties decoding the emotional meanings in facial expressions were found to associated with a poor ability to have positive interpersonal relationships (22). In addition, a number of studies have been conducted in the last decades on the relationship between emotion recognition abilities and interpersonal problems in samples suffering from mental or neurological disorders. In this research, the Inventory of Interpersonal Problems (IIP) (23, 24) was used, a widespread self-report questionnaire to assess interpersonal impairment in clinical contexts.

Kornreich et al. (25) observed that detoxified patients suffering from alcohol use disorder had deficits in the identification of facial emotions, which were associated with the global severity of patients’ interpersonal problems. In a later study (26), a specific association between difficulties in the recognition of facial anger and global severity of interpersonal problems was reported in abstinent individuals with alcohol use disorders. In a sample of children with attention-deficit hyperactivity disorder (ADHD) there was a strong negative relation between severity of interpersonal problems and overall decoding accuracy for facial emotions (27). Interestingly, the correlation between interpersonal difficulties and accuracy in decoding emotions was highest for facial anger. Finally, in a sample of patients with Parkinson’s disease deficiencies in facial emotion recognition were also associated with higher levels of interpersonal problems (28). In summary, there is evidence for an association between deficits in the ability to recognize facial emotions and interpersonal difficulties across different disorders. It appears that in particular difficulties in recognizing threat-related or negative emotions (such as anger) could be related to interpersonal problems. In interpersonal communication, facial expressions of anger signal goal obstruction and represent signs of potential aggression and proneness to engage in a conflict (16, 29). Deficits in anger recognition may lead to less responsivity and reduced search for the causes on the part of the observer. In the long term, partners could be increasingly dissatisfied with such relationships as they may feel not being taken seriously in their actual needs and intentions.

To our knowledge, no research has investigated the identification of facial emotions as a function of the interpersonal styles agency and communion. A recent study on psychopathic traits (30) provides an indication how a disposition to dominate and control others could affect perception of facial emotions: social dominance and lack of anxiety were found to be linked to decreased fear and anger recognition in facial expressions. Moreover, it can be assumed that the interpersonal dimension of communion should be positively associated with the recognition of emotions from others’ facial expressions. It can be argued that individuals characterized by high communion are strongly touched by the distress of other people and often worry about others’ problems. They empathize with others. According to the results of a meta-analysis (31), there exist significant positive relations between affective empathy and facial emotion perception as well as between cognitive empathy and facial emotion perception.

The present study examined whether associations exist between interpersonal problems and emotion recognition deficits independently from the presence of a mental or neurological disorder in healthy adults. The presence of a clinical disorder could have an effect on severity and type of interpersonal problems as well as on severity and type of deficits in facial emotion recognition and modify its relationship as a function of the specific disorder. To this aim, the Inventory of Interpersonal Problems (32) and an emotion recognition task assessing decoding accuracy for six emotional facial expressions (happiness, surprise, anger, disgust, sadness, and fear) presented in frontal and in profile view were administered to healthy individuals. Recently, comprehensive analyses of responses on the IIP based on large samples from different countries and cultural backgrounds indicate that the latent structure of interpersonal problems is best represented by two continuous dimensions, which are largely independent of each other, i.e., agency and communion (33). Therefore, we focused our analyses on these two interpersonal dimensions. There are a number of other relevant variables such as negative affects, verbal intelligence, and gender that can affect interpersonal functioning as well as emotion recognition and should be taken into consideration when examining the relationship between interpersonal and emotion recognition difficulties.

Negative affects frequently accompany experiences of interpersonal failure and may further intensify relationship problems (34, 35). Likewise negative affect such as anxiety or depressed mood can have an impact on facial emotion recognition (36, 37). Verbal intelligence is an additional factor influencing labeling and recognition of emotions in facial expressions (38, 39). Finally, gender is also a variable that plays a role in the present research context, as women have been found to describe themselves as more caring and less assertive compared to men (40, 41) and to recognize facially expressed emotions better than men (42).

Based on the results from previous studies that analyzed the effects of social dominance (30) and empathy (31) on facial emotion identification we expected that the interpersonal dimension of agency would be negatively related to threat-related emotions (i.e., anger, fear, and disgust). Moreover, we hypothesized that the interpersonal dimension of communion would be positively associated with the recognition of emotions from others’ facial expressions.

## Materials and methods

### Participants

Our study participants were recruited *via* public notices and online advertisements. The final sample consisted of 190 young healthy individuals (95 women, 95 men) with a mean age of 23.9 years (*SD* = 3.8; range: 18–35). All participants were native speakers of German and had normal or corrected-to-normal vision as tested with a Snellen eye chart. Their mean duration of school education was 12.2 years (*SD* = 0.7). Most participants were university students (80%). At the start of the study, after informing about the study and the procedure participants were interviewed *via* telephone by trained doctoral students about their mental health status and relevant hospitalizations and treatments. Exclusion criteria for study participation were actual or past presence of mental or neurological disorders (mental health problems, neurological problems, psychiatric treatments and hospitalizations, neurological treatments, psychotherapies, current use of psychotropic medication) and current moderate or severe depressive symptoms (as assessed by the BDI-II (score ≥ 20)). The interviewers were instructed, trained, and supervised by an experienced clinical psychologist. The interview and testing sessions took place on different days. All participants were financially compensated for their involvement in the study.

### Measures

The German version of the *Inventory of Interpersonal Problems* (IIP-D) (32) consists of 64 items as the original version of the IIP (24). The IIP-D has eight items for each of its eight subscales: Domineering [i.e., difficulties in relaxing control over others (PA)], Vindictive [i.e., difficulties of hostile dominance and the tendency to fight with others (BC)], Cold [i.e., low degrees of affection for and connection with others (DE)], Socially Avoidant [i.e., feelings of anxiety and avoidance in the presence of others (FG)], Nonassertive [i.e., difficulties in taking initiative in relation to others and coping with social challenges (HI)], Exploitable [i.e., excesses of friendly submissiveness (JK)], Overly Nurturant [i.e., tendency to affiliate excessively (LM)], and Intrusive [i.e., problems with friendly dominance (NO)]. The inventory uses a 5-point scale ranging from 0 (not at all) to 4 (extremely) corresponding to different distressing interpersonal excesses or inhibitions. The IIP is a widely used clinical and research measure of interpersonal difficulties with strong psychometric properties (24, 32, 43, 44). In the present sample, Cronbach’s alphas for the IIP scales indicated an acceptable to good level of reliability [Domineering (PA): 0.69, Vindictive (BC): 0.60, Cold (DE): 0.76, Socially avoidant (FG): 0.80, Nonassertive (HI): 0.82, Exploitable (JK): 0.77, Overly nurturant (LM): 0.71, and Intrusive (NO): 0.71]. In our study, we followed the scoring procedure for the two interpersonal dimensions as suggested by Wendt et al. (33):

$\begin{array}{c} Agency=PA+\left(NO\times 0.71\right)+\left(BC\times 0.71\right)-\left(FG\times 0.71\right)\\ -\left(JK\times 0.71\right)-HI. \end{array}$

$\begin{array}{c} Communion=LM+\left(NO\times 0.71\right)+\left(JK\times 0.71\right)-\left(BC\times 0.71\right)\\ -\left(FG\times 0.71\right)-DE. \end{array}$

The *State–Trait Anxiety Inventory* [STAI; German version (45)] is a commonly used self-report measure of trait and state anxiety. It consists of 20 questions, respectively, that are evaluated on a 4-point Likert scale. The state version of the STAI assesses the level of anxious feelings at the moment, whereas the trait version measures relatively stable interindividual differences in evaluating and experiencing situations as threatening. Cronbach’s alphas for the STAI state and STAI trait were 0.80 and 0.90.

The *Beck Depression Inventory* is a 21-question multiple-choice self-report scale [BDI-II; German version (46)] that assesses the severity and presence of depressive symptoms such as hopelessness, irritability, negative cognitions as well as physical symptoms during the preceding 2 weeks. Each item is scored on a scale from 0 to 3 based on a list of four statements arranged in increasing severity about a particular symptom of depression. Based on the standardized cutoff values of the BDI-II level of depression can be interpreted as minimal (0–13), mild (14–19), moderate (20–28), or severe (≥29). In the present sample, Cronbach’s alpha for the BDI-II was 0.87.

The *Multiple-choice vocabulary intelligence test* [Mehrfachwahl-Wortschatz-Intelligenztest, MWT-B (47)] is a performance test that measures aspects of general intelligence, specifically crystallized, verbal intelligence. The MWT-B includes 37 items. Each item consists of one real word and four pronounceable pseudo-words (e.g., Funktion–Kuntion–Finzahm–Tuntion–Tunkion). Subjects are asked to find the real word and to underline it. Each word correctly recognized gives a point. There are no time restrictions. Raw scores can be converted to IQ scores.

### Emotion recognition task: Stimuli and procedure

Our recognition task was constructed on the basis of the Karolinska Directed Emotional Faces (KDEF) (48). Facial stimuli consisted of 140 color photographs of 10 models (five female, five male), Caucasian amateur individuals, chosen from the KDEF. Each model posed seven different facial expressions (happy, surprised, angry, sad, fearful, disgusted, and neutral) at two different viewing angles: full-face frontal view, and a left profile view. The display size of each face photo on the screen was 14.5 cm high × 14.2 cm wide.

The experiment started with 14 practice trials in which all presentation conditions (7 expressions × 2 viewing angles) were shown once. In the practice trials, different models were presented. Each trial had the following routine: after the presentation of a fixation cross for 800 ms, a facial expression was presented for 700 ms. After presentation of the face, participants had to label the facial emotions by button presses in a forced choice manner without a time limit. Participants gave responses on a keyboard using the number keys 1 (happiness), 2 (surprise), 3 (anger), 4 (disgust), 5 (anxiety), 6, (sadness), or 7 (neutral). Each emotion was assigned to one key during the entire experiment. After the target stimulus, the expression categories and the assigned numbers were shown at the bottom of a black screen in white letters until a response was given. The intertrial interval had a duration of 2,000 ms. Participants were instructed that they would see photos of faces expressing the emotions happiness, surprise, anger, disgust, anxiety, or sadness. Moreover, they were informed that some faces would have a neutral expression and that some faces would be seen in frontal view, others from the side. Participants were instructed to identify the expression of each face and to respond as accurate as possible. Participants took a short break after 50 and 100 trials. Trials were shown in a fixed random sequence with the constraints that no two subsequent trials depict the same person, and not more than three subsequent trials show the same emotion. Participants did not receive feedback whether their responses were correct or incorrect. During the experiment participants were seated in a chair at approximately 60 cm in front of the screen. The computer-based stimulus presentation and response registration were realized *via* Inquisit (49) on a Dell Latitude E6510 with a 15.6-inch screen. Emotion recognition accuracy was assessed using the unbiased hit rate as proposed by Wagner (50). The unbiased hit rate expresses accuracy as proportions of both response frequency and stimulus frequency and is insensitive to bias, to proportions of stimuli of different types, and to the number of categories. The unbiased hit rate can vary between 0 and 1 (50).

### Procedure

The experiment took place at the Department of Psychosomatic Medicine and Psychotherapy at the University of Leipzig. All subjects were tested individually in a quiet room. Due to the COVID-19 pandemic, all participants and the experimenter wore a face mask throughout the experiment. At the beginning of the study, participants completed a sociodemographic questionnaire and performed a vision test (using the Snellen eye chart). The tests and questionnaires were administered in the following fixed order: STAI state, STAI trait, MWT-B, BDI-II, IIP. At the end of the session, participants were given the emotion recognition task.

### Statistical analyses

Product–moment correlation analyses were performed to examine the relationships between IIP dimensions, measures of negative affectivity, verbal intelligence, and recognition of emotional facial expressions. Correlation analyses between IIP scales and emotion recognition performance were primarily performed to better understand how the poles of the interpersonal dimensions contribute to significant correlations at the dimensional level. To investigate gender differences concerning interpersonal problems independent samples *t*-tests were performed. Data of the emotion recognition task were analyzed by a repeated measures ANOVA with the factor emotion (happiness, surprise, anger, disgust, fear, sadness, and neutrality). Greenhouse–Geisser correction (51) was used to adjust the degrees of freedom of the F-ratios when the assumption of sphericity was violated. Follow-up tests were conducted to evaluate pairwise differences (Bonferroni-adjusted pairwise comparisons). In addition, hierarchical regression analyses were conducted for IIP dimensions, which showed correlations with recognition of facial emotions, to examine whether these relationships remain significant after adjusting the effects of other relevant variables such as gender, and negative affectivity of participants. Results were considered significant at *p* < 0.05, two-tailed. All calculations were administered using SPSS 27.0 (IBM Corp., Armonk, NY, United States).

## Results

### Relationships of interpersonal problems with psychological measures

Descriptive statistics for self-report scales and MWT-B are shown in Table 1. Women had lower agency [−13.65 (*SD* = 11.50)] and higher communion scores [11.59 (*SD* = 12.51)] compared to men [−7.84 (*SD* = 12.13) and 7.86 (*SD* = 11.26)], *t*(188) = −3.39, *p* = 0.001; and *t*(188) = 2.16, *p* < 0.05. Correlation analyses revealed that interpersonal agency was negatively associated with trait anxiety (*r* = −0.28, *p* < 0.001) but not related to state anxiety, depressive symptoms, and verbal intelligence. Interpersonal communion was not correlated with state and trait anxiety, depressive symptoms, and verbal intelligence.

**Table 1:** Descriptive statistics for psychological measures.

Variable	Mean	*SD*
IIP domineering	6.08	4.10
IIP vindictive	7.05	3.58
IIP cold	8.22	5.08
IIP socially avoidant	9.61	5.42
IIP nonassertive	12.77	5.79
IIP exploitable	13.24	5.43
IIP overly nurturant	13.22	5.00
IIP intrusive	10.08	4.91
IIP agency (dimension)	−10.74	12.14
IIP communion (dimension)	9.73	12.02
MWT-B IQ	110.22	11.20
STAI state	34.56	6.06
STAI trait	38.55	8.45
BDI-II	7.29	5.03

IIP, Inventory of Interpersonal Problems; MWT-B, Multiple-choice vocabulary test version B; intelligence quotient; STAI, State Trait Anxiety Inventory; BDI-II, Beck Depression Inventory.

### Emotion recognition

Participants’ recognition performance is presented in Table 2. A repeated measures ANOVA based on unbiased hit rates for facial expressions yielded a significant effect of emotion, *F*(4.58, 866.54) = 466.74, *p* < 0.001, η*_p_*^2^ = 0.71. Bonferroni-adjusted pairwise comparisons showed that facial expressions of happiness were better recognized than all other expressions, whereas recognition of fearful faces was significantly worse than for all other emotions (*p* < 0.001, respectively). Moreover, neutral expression was better recognized than anger; anger better than disgust; disgust better than sadness and surprise (*p*s < 0.01). Recognition of facial expressions of sadness and surprise did not differ from each other. There were no differences in emotion identification between women and men, except for sad facial expressions: women had a higher hit rate for facial sadness [0.65 (*SD* = 0.14)) than men (0.60 (*SD* = 0.17)], *t*(188) = 2.06, *p* < 0.05.

**Table 2:** Unbiased hit rates for emotional facial expressions in the emotion recognition task (means with standard deviations).

Facial expression	Mean	*SD*
Happiness	0.91	0.08
Surprise	0.62	0.10
Anger	0.73	0.16
Disgust	0.69	0.14
Sadness	0.63	0.15
Fear	0.34	0.17
Neutral	0.77	0.15

### Relationships of emotion recognition with interpersonal problems, negative affectivity, and verbal intelligence

Interpersonal agency was negatively related to the hit rate for surprised, angry, and disgusted facial expressions (see Table 3). In contrast, interpersonal communion showed no correlations with emotion recognition performance (see Table 3). State anxiety was negatively correlated with the hit rate for happy, and disgusted facial expressions (*r* = −0.18 and −0.16, *p*s < 0.05), whereas trait anxiety was positively associated with the hit rate for surprised faces (*r* = 0.17, *p* < 0.05). Level of depressive symptoms and verbal intelligence were not correlated with emotion recognition performance. A correlation analysis between IIP scales and emotion recognition showed significant negative correlations of the scales domineering (PA), intrusive (NO), and vindictive (BC) with identification of angry and disgusted facial expressions (see Table 4). The IIP scales nonassertive (HI), exploitable (JK), and socially avoidant (FG) did not correlate with identification of angry and disgusted faces or the recognition of other facial expressions (see Table 4). Interestingly, the IIP scale nonassertive (HI) was positively associated with the recognition of facial surprise.

**Table 3:** Correlations between interpersonal dimensions (IIP) and recognition of emotional facial expressions (unbiased hit rates).

Facial expression	IIP agency	IIP communion
Happiness	−0.03	−0.01
Surprise	−0.14*	0.10
Anger	−0.19**	0.01
Disgust	−0.19**	−0.08
Sadness	−0.05	0.05
Fear	−0.11	0.01
Neutral	−0.06	−0.02

*p < 0.05; **p < 0.01 (two-tailed).

**Table 4:** Correlations between IIP scales and recognition of emotional facial expressions (unbiased hit rates).

Facial expression	PA	BC	DE	FG	HI	JK	LM	NO
Happiness	0.01	−0.19*	−0.02	−0.08	−0.04	−0.01	−0.09	−0.15^
Surprise	−0.04	−0.14	−0.04	0.01	0.15^	0.08	0.08	−0.01
Anger	−0.29**	−0.30**	−0.18^	−0.11	0.02	−0.04	−0.19*	−0.25**
Disgust	−0.22*	−0.16^	−0.08	−0.01	0.06	−0.03	−0.17^	−0.23*
Sadness	−0.15^	−0.21*	−0.13	−0.07	−0.07	−0.02	−0.11	−0.07
Fear	−0.16^	−0.19*	−0.03	−0.12	0.02	0.00	−0.07	−0.17^
Neutral	−0.20*	−0.21*	−0.12	−0.07	−0.10	−0.09	−0.15^	−0.16^

^*p* < 0.05; *p < 0.01; **p < 0.001 (two-tailed). PA, Domineering; BC, Vindictive; DE, Cold; FG, Socially Avoidant; HI, Nonassertive; JK, Exploitable; LM, Overly Nurturant; NO, Intrusive.

A regression model for interpersonal agency was calculated to examine whether the hit rate for facial surprise is a predictor independent from gender, and negative affectivity. In the first step of the hierarchical regression analysis, variance in agency was significantly explained by gender, with men showing higher values on agency (see Supplementary Table 1). In step two entering the STAI state, STAI trait, and BDI-II, the STAI trait significantly increased the predictive value of the model. This means, trait anxiety was found to be a negative predictor of interpersonal agency. Unbiased hit rate for facial surprise did not significantly predict agency in step three (see Supplementary Table 1).

We calculated an additional regression model for agency with hit rate for facial anger entered in the third step as predictor. Unbiased hit rate for facial anger was a significant negative predictor of interpersonal agency (see Supplementary Table 2). Finally, a regression model was calculated for agency with hit rate for facial disgust entered in the third step as predictor. Unbiased hit rate for facial disgust was a significant negative predictor of interpersonal agency (see Supplementary Table 3).

## Discussion

The present study was conducted to investigate whether there are associations between interpersonal problems and deficits in the ability to correctly recognize emotions from facial expressions in healthy adults. Thus, our study was focused on facial emotion recognition accuracy, which represents a crucial part of successful social interactions. For our purpose, we presented six qualities of emotional facial expressions in frontal and in profile view along with the Inventory of Interpersonal Problems (IIP) (32) to 190 healthy men and women. In our study, unbiased hit rates (50) were analyzed in order to ensure that recognition accuracy was not influenced by response bias effects. Our analysis was focused on the two main dimensions of interpersonal problems, i.e., agency, and communion (33). An important factor contributing to difficulties in interpersonal behavior could be an impaired identification of emotions from other people’s facial expressions (6).

According to our results, interpersonal agency was negatively related to the recognition of anger and disgust in facial expressions, confirming in part our hypothesis. These relations were independent of participants’ gender, and negative affectivity. This means that difficulties of hostile social dominance, problems in taking care of other people’s needs, and an exaggerated desire to control others go along with a reduced ability to identify facial anger and disgust. It should be noted that the size of the observed correlations indicates small to medium relationships between interpersonal agency and poor recognition of anger and disgust. Thus, even if it is correct to assume that deficits in threat decoding represent a factor contributing to interpersonal problems related to agency one clearly has to consider other relevant factors in this context such as, for example, the ability to manage conflicts or to regulate one’s emotions (52).

Missing signals of other people’s anger and disgust might be partly responsible for interpersonal problems with dominance and intrusiveness. Missing or incorrect interpretation of facial signals of anger could be especially relevant to generate and intensify interpersonal conflict. According to Delk et al. (30) anger recognition helps to assess the effect of one’s actions on others and if necessary to halt them. In case of anger recognition deficits, the risk should increase that others engage in more aggressive interactions leading to higher incidence of mutual retaliation and damage (30). As noted in the introduction, facial anger signals goal obstruction and proneness to engage in conflict and aggressive behavior (16, 29). An impaired decoding of anger could lead to less response on part of the observer and might have long-term adverse effects on relationships by potentially escalating and prolonging conflicts. Partners may feel not being taken seriously in their needs and intentions. The present finding is consistent with and extends results from clinical studies demonstrating a negative association between severity of interpersonal problems and recognition of facial anger in childhood ADHD (27) and a negative relationship of severity of interpersonal problems and, in particular, the IIP scale Intrusive with anger processing accuracy in alcohol use disorder (26). Thus, there is preliminary evidence that interpersonal problems and especially those related to agency could be linked to poor decoding of facial anger independently from the presence of a mental disorder.

Our data indicate that excessive tendencies to dominate and control others are also linked to a decreased recognition of disgust in others’ facial expressions. Disgust is a hostility-related emotion associated with aggression and conflict (53) that expresses disapproval for the actions of other people (54) and that motivates avoidance and withdrawal behavior (55, 56). In social interactions, facial expressions of disgust signal revulsion and interpersonal rejection (17) and indicate a request to increase interpersonal distance (13). Missing or misidentifying disgust expressions of interaction partners individuals high on agency could continue to exhibit intrusive behaviors and, in this way, further escalate interpersonal conflicts.

It is noteworthy that the facial expressions of anger and disgust were not among the most difficult to recognize in our experiment. Thus, interpersonal agency was not found to be related to expression conditions, which were particularly difficult to identify. Performance in our emotion recognition task showed that happiness was the best and fear the worst recognized emotion across view conditions (frontal and profile). Hit rates for neutrality, anger, disgust, surprise, and sadness were in between. These recognition rates are comparable to those of previous studies (57–60) although in our task facial expressions were shown for only 700 ms. As correlation does not imply causation longitudinal studies are necessary, including for example a training of anger recognition, to draw conclusions about causal effects of recognition deficits on experiences of interpersonal agency. In general, to get more insight into the temporal and causal relationships among interpersonal dysfunctions and facial emotion recognition it could be helpful to develop paradigms that combine performance based and ecological assessment methods that measure social behaviors and emotion recognition with high temporal resolution and ecological validity in interpersonal situations (61). Traditional approaches of emotional and social cognition research based on single experimental sessions have been challenged by shifting the focus to the interpersonal situation and the iterative changes in perceptions and social behavior, which occur in real-time and across repeated interactions (62).

The present data did not support the hypothesis that the interpersonal dimension of communion is positively associated with the recognition of emotions from others’ facial expressions. We found no correlations of communion with emotion recognition performance in our investigation. This means that individuals with a tendency to affiliate excessively seem no better and no worse in recognizing others’ facial emotions than individuals with a low tendency to affiliate. Our findings are somewhat at odds with the conclusion of a recent meta-analysis (31) that reported positive but weak relations of affective and cognitive empathy with the ability to recognize facial emotions. However, it should be noted that non-clinical empathy and interpersonal problems in communion, which refer to overinvolvement with others and difficulties to maintain personal boundaries, differ at least in part in their constructs.

An important task of future research on emotion perception and interpersonal problems could be the study of negative biases in the perception of neutral (or ambiguous) facial expressions as a function of interpersonal difficulties. Possibly, individuals who are excessively vindictive and mistrustful of others may tend to misattribute negative emotions such as anger or disgust to neutral expressions of other people. Findings from clinical research suggest that individuals suffering, for example, from schizophrenia-spectrum or borderline personality disorders exhibit negatively biased interpretations of neutral faces (63, 64).

Some limitations have to be acknowledged in the evaluation of our results. First, our sample consisting of young and well-educated individuals is not representative of the general adult population. This limits the generalizability of our results. It is necessary to replicate our findings in populations other than college students. In our emotion recognition task, we administered emotional facial expressions at full intensity but did not use more subtle expressions of emotions, which are more difficult to decode. It remains to be clarified in future research whether interpersonal problems could be related more strongly to the recognition of low intensity expressions. Another limitation is that static stimuli (photographs) were shown in our study, yet emotional facial expressions in daily life are in general dynamic. The mechanisms underlying the association between deficits in emotion recognition and interpersonal problems remain to be further clarified. Registration of eye-gaze during emotion recognition tasks in future investigations could help to specify our understanding of the mechanisms underlying poor facial emotion recognition in individuals with interpersonal problems. It would be important to know whether individuals suffering from interpersonal agency look at anger and disgust faces (and their diagnostically relevant facial features) more briefly or in a different way compared to individuals without these problems. Tendencies to avoid fixating and exploring angry (or disgusted) facial expressions could become a target of intervention and could have implications for the constructions of training programs intended to improve emotion recognition. Future intervention studies may clarify whether individuals suffering from problems with interpersonal agency may benefit from training programs targeted to enhance the ability to identify anger and disgust in facial expressions. The use of a dimensional approach with the computation of scores for agency and communion seems to be promising in future clinical studies on interpersonal problems and emotion recognition.

## Data availability statement

The raw data supporting the conclusions of this article will be made available by the authors, without undue reservation.

## Ethics statement

The studies involving human participants were reviewed and approved by the Ethics Committee at the University of Leipzig, Medical Faculty. The patients/participants provided their written informed consent to participate in this study.

## Author contributions

TS and AK developed the research idea and designed the study. AL involved in data collection. TS analyzed the data and drafted the manuscript. KK, AK, and AL contributed substantially to data interpretation and revised critically the manuscript. All authors read and approved the final version of the manuscript.

## Conflict of interest

The authors declare that the research was conducted in the absence of any commercial or financial relationships that could be construed as a potential conflict of interest.

## Publisher’s note

All claims expressed in this article are solely those of the authors and do not necessarily represent those of their affiliated organizations, or those of the publisher, the editors and the reviewers. Any product that may be evaluated in this article, or claim that may be made by its manufacturer, is not guaranteed or endorsed by the publisher.

## Funding

The authors acknowledge support from the German Research Foundation (DFG) and Universität Leipzig within the program of Open Access Publishing.
