# Supplementary material for: Interpersonal problems and recognition of facial emotions in healthy individuals
Source: Front Psychiatry. 2023 Apr 17;14:1139051. doi: 10.3389/fpsyt.2023.1139051 (PMC10149975; doi:10.3389/fpsyt.2023.1139051)
Supplement: Supplementary file 1 [file Table_1.DOCX]

Supplementary Table 1: Hierarchical regression predicting the interpersonal dimension agency in three steps by gender, state anxiety (STAI), trait anxiety (STAI), depression (BDI-II), and recognition of facial surprise (unbiased hit rate) (N = 190).

|  | **Coefficients Multicollinearity Model** | | | | | | | |
| --- | --- | --- | --- | --- | --- | --- | --- | --- |
| **Predictor** | **β** | **Beta** | ***t*** | **Sig. (*p*)** | **Tol.** | **VIF** | **R^2^** | ∆**R^2^** |
| **Step1** Gender | 5.81 | .24 | 3.39 | .001 | 1.00 | 1.00 | .058** | - |
| **Step2** State anxiety | -0.05 | -.03 | -0.33 | .74 | .72 | 1.38 | .124** | .066* |
| Trait anxiety | -0.40 | -.28 | -3.10 | .002 | .58 | 1.72 |  |  |
| Depression | 0.19 | -.08 | -0.93 | .353 | .68 | 1.47 |  |  |
| **Step 3**  Recognition  surprise | -10.36 | -.08 | -1.20 | .233 | .93 | 1.07 | .131** | .007 |

Gender code: 1 = female, 2 = male; β = unstandardized regression coefficient; Tol. = Tolerance; VIF = Variance Inflation Factor.

* *p* <.01; ** *p* ≤ .001.

Supplementary Table 2: Hierarchical regression predicting the interpersonal dimension agency in three steps by gender, state anxiety (STAI), trait anxiety (STAI), depression (BDI-II), and recognition of facial anger (unbiased hit rate) (N = 190).

|  | **Coefficients Multicollinearity Model** | | | | | | | |
| --- | --- | --- | --- | --- | --- | --- | --- | --- |
| **Predictor** | **β** | **Beta** | ***t*** | **Sig. (*p*)** | **Tol.** | **VIF** | **R^2^** | ∆**R^2^** |
| **Step1** Gender | 5.81 | .24 | 3.39 | .001 | 1.00 | 1.00 | .058** | - |
| **Step2** State anxiety | -0.05 | -.03 | -0.33 | .74 | .72 | 1.38 | .124** | .066* |
| Trait anxiety | -0.40 | -.28 | -3.10 | .002 | .58 | 1.72 |  |  |
| Depression | 0.19 | -.08 | -0.93 | .353 | .68 | 1.47 |  |  |
| **Step 3**  Recognition  anger | -13.95 | -.18 | -2.67 | .008 | .97 | 1.03 | .156** | .033* |

Gender code: 1 = female, 2 = male; β = unstandardized regression coefficient; Tol. = Tolerance; VIF = Variance Inflation Factor.

* *p* <.01; ** *p* ≤ .001.

Supplementary Table 3: Hierarchical regression predicting the interpersonal dimension agency in three steps by gender, state anxiety (STAI), trait anxiety (STAI), depression (BDI-II), and recognition of facial disgust (unbiased hit rate) (N = 190).

|  | **Coefficients Multicollinearity Model** | | | | | | | |
| --- | --- | --- | --- | --- | --- | --- | --- | --- |
| **Predictor** | **β** | **Beta** | ***t*** | **Sig. (*p*)** | **Tol.** | **VIF** | **R^2^** | ∆**R^2^** |
| **Step1** Gender | 5.81 | .24 | 3.39 | .001 | 1.00 | 1.00 | .058** | - |
| **Step2** State anxiety | -0.05 | -.03 | -0.33 | .74 | .72 | 1.38 | .124** | .066* |
| Trait anxiety | -0.40 | -.28 | -3.10 | .002 | .58 | 1.72 |  |  |
| Depression | 0.19 | -.08 | -0.93 | .353 | .68 | 1.47 |  |  |
| **Step 3**  Recognition  disgust | -15.34 | -.18 | -2.64 | .009 | .96 | 1.04 | .156** | .032* |

Gender code: 1 = female, 2 = male; β = unstandardized regression coefficient; Tol. = Tolerance; VIF = Variance Inflation Factor.

* *p* <.01; ** *p* ≤ .001.
